# Bioelectrical impedance analysis in clinical practice: implications for hepatitis C therapy BIA and hepatitis C

**DOI:** 10.1186/1743-422X-7-191

**Published:** 2010-08-16

**Authors:** Alisan Kahraman, Johannes Hilsenbeck, Monika Nyga, Judith Ertle, Alexander Wree, Mathias Plauth, Guido Gerken, Ali E Canbay

**Affiliations:** 1 University Clinic Duisburg-Essen, Department of Gastroenterolgy and Hepatology, Hufelandstrasse 55, 45122 Essen, Germany; 2 Krankenhaus Dueren gem. GmbH, Internal Medicine II, Roonstr. 30, 52351 Dueren, Germany; 3 Städtisches Klinikum, Department of Internal Medicine, Auenweg 38, 06847 Dessau, Germany

## Abstract

**Background:**

Body composition analysis using phase angle (PA), determined by bioelectrical impedance analysis (BIA), reflects tissue electrical properties and has prognostic value in liver cirrhosis. Objective of this prospective study was to investigate clinical use and prognostic value of BIA-derived phase angle and alterations in body composition for hepatitis C infection (HCV) following antiviral therapy.

**Methods:**

37 consecutive patients with HCV infection were enrolled, BIA was performed, and PA was calculated from each pair of measurements. 22 HCV genotype 3 patients treated for 24 weeks and 15 genotype 1 patients treated for 48 weeks, were examined before and after antiviral treatment and compared to 10 untreated HCV patients at 0, 24, and 48 weeks. Basic laboratory data were correlated to body composition alterations.

**Results:**

Significant reduction in body fat (BF: 24.2 ± 6.7 kg vs. 19.9 ± 6.6 kg, genotype1; 15.4 ± 10.9 kg vs. 13.2 ± 12.1 kg, genotype 3) and body cell mass (BCM: 27.3 ± 6.8 kg vs. 24.3 ± 7.2 kg, genotype1; 27.7 ± 8.8 kg vs. 24.6 ± 7.6 kg, genotype 3) was found following treatment. PA in genotype 3 patients was significantly lowered after antiviral treatment compared to initial measurements (5.9 ± 0.7° vs. 5.4 ± 0.8°). Total body water (TBW) was significantly decreased in treated patients with genotype 1 (41.4 ± 7.9 l vs. 40.8 ± 9.5 l). PA reduction was accompanied by flu-like syndromes, whereas TBW decline was more frequently associated with fatigue and cephalgia.

**Discussion:**

BIA offers a sophisticated analysis of body composition including BF, BCM, and TBW for HCV patients following antiviral regimens. PA reduction was associated with increased adverse effects of the antiviral therapy allowing a more dynamic therapy application.

## Background

Bioelectrical impedance analysis (BIA) has been introduced as a non-invasive, rapid, easy to perform, reproducible, and safe technique for the analysis of body composition [1]. It is based on the assumption that an electric current is conducted well by water and electrolyte-containing parts of a body but poorly by fat and bone mass. A fixed, low-voltage, high-frequency alternating current introduced into the human body or tissue is conducted almost completely through the fluid compartment of the fat-free mass [2]. BIA measures parameters such as resistance (R) and capacitance (Xc) by recording a voltage drop in applied current [3]. Capacitance causes the current to lag behind the voltage, which creates a phase shift. This shift is quantified geometrically as the angular transformation of the ratio of capacitance to resistance, or the phase angle (PA) [4]. PA reflects the relative contribution of fluid (resistance) and cellular membranes (capacitance) of the human body. By definition, PA is positively associated with capacitance and negatively associated with resistance [4]. PA can also be interpreted as an indicator of water distribution between the extra- and intracellular space, one of the most sensitive indicators of malnutrition [5,6].

BIA-derived PA could serve as prognostic marker in several clinical conditions where cell membrane integrity is compromised and alterations in fluid balance are noted, such as malnutrition in advanced neoplastic diseases or decompensated liver cirrhosis [2,7-21]. However, there are no data on body composition in patients with HCV infection before and after antiviral treatment which is an important factor for treatment decisions, especially if supplemental therapy is needed. Indeed, interferon-α (IFN-α) and ribavirin treatment in HCV is often associated with fatigue, cephalgia, weight loss, flu-like syndromes, and anorexia [22], implying changes in nutritional status and body composition [23].

## Objective

The primary objective of the present study was to prospectively evaluate effects of antiviral therapy on BIA-derived PA as a simple method for the estimation of body cell mass (BCM), body fat (BF), extracellular mass (ECM), and total body water (TBW) in 37 patients with chronic HCV infection.

## Study Design

### Patient population

The study was performed on a consecutive case series of 37 patients with chronic HCV infection (October 2008 - September 2009). Inclusion criteria were age ≥ 18 years, chronic HCV infection, and a liver biopsy performed within the last 6 months. Exclusion criteria included decompensated liver disease, peripheral oedema, pre-existent malnutrition, decreased albumin levels (< 3.4 g/dl), hepatocellular carcinoma (HCC), active alcohol abuse, co-infection with HBV or HIV, chronic renal failure (GFR < 50 ml/min./1.73 m^2^), and overt diabetes. Treated patients were divided into 2 groups according to HCV genotype and duration of antiviral therapy. All patients underwent baseline laboratory measurements. Full written informed consent was obtained from all subjects before entry into the study, and the clinic's ethics committee approved the protocol. All of the treated HCV patients received pegylated interferon-α (1.5 mg/kg body weight weekly s.c.) and ribavirin (12 mg/kg body weight daily p. o.) as antiviral therapy and completed the 24 or 48 week cycle with the starting dose. Patients with the need of dose adjustment were excluded in order to avoid effects of the dose on alterations in body composition. In addition, none of the included patients needed supportive medication with granulokine or epo. Moreover, no patient received other antiviral or steatosis-inducing drugs. Occurrence and severity of side effects was monitored by a study nurse who was blinded to the results of BIA measurements.

### Virology

All HCV patients had a positive anti-HCV status (CMIA anti-HCV, Abbott Laboratories, Wiesbaden, Germany), positive HCV-RNA in serum, and increased liver enzymes. HCV genotyping was performed with INNO-LIPA HCV II kits (Siemens Healthcare Diagnostics, Marburg, Germany) according to the manufacturer's instructions. Amplicor-HCV-Monitor (Perkin-Elmer, Norwalk, Connecticut, USA) was used to quantify HCV-RNA levels in serum. The detection limit was < 615 copies/ml.

### BIA measurement procedures

**Figure 1:**
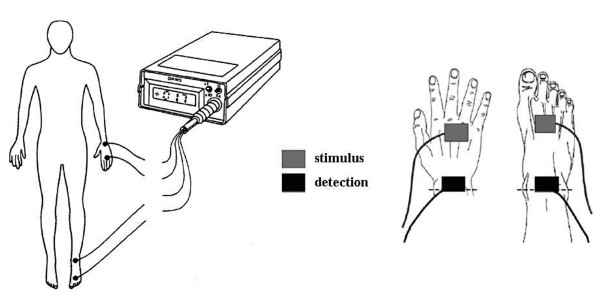
**Schematic representation of BIA measurements using signal and detection electrodes**.

BIA was performed by a registered study nurse (M. N.). Impedance measurements were taken after 10 minutes of rest with a BIA impedance analyzer (BIA 101, Akern Bioresearch, Florence, Italy). Briefly, two pairs of electrodes were attached on the right hand and right foot with the patient in supine position, with legs slightly apart, and the arms not touching the torso [4] (Figure 1). Calculation of TBW, BF, and BCM was performed as previously described elsewhere [24-26].

### Statistical analysis

Statistical analysis was performed using the SPSS 11.5 system (SPSS Incorporation, Chicago, Illinois, USA). Continuous variables are presented as means ± standard deviation (SD) whereas categorical variables are presented as count and proportion. Comparison between groups were made using the Mann-Whitney U test or the Student's test for continuous variables, and the χ^2 ^or Fisher's exact probability test for categorical data. A p-value < 0.05 was considered to be statistically significant. Multiple comparisons between more than two groups of patients were performed by ANOVA and subsequent least-significant difference procedure test. Spearman's correlation coefficient was calculated for testing the relationship between different quantities in a bivariate regression model.

## Results

### Patients' demographic data

Table 1 shows the baseline characteristics of 37 patients with chronic HCV infection and 10 therapy-naïve subjects with HCV infection (5 with genotype 1 and 5 with genotype 3). Genotype 1 was present in 15 patients (8 males, 7 females, mean age 48.1 ± 12.6 y) whereas 22 patients had genotype 3 (10 males, 12 females, 37.5 ± 9.5 y). Patients with genotype 3 were treated for 24 weeks whereas subjects with genotype 1 received antiviral therapy for 48 weeks. Virological response was observed in 73.3% of patients with genotype 1 and in 86.3% with genotype 3. In addition, we also performed ultrasound examinations to exclude ascites and used the FibroScan to measure extent of liver fibrosis. However, we found no positive correlation between BIA measurements and liver stiffness (data not shown).

**Table 1:** Baseline biochemical and physical characteristics of the study populations.

	HCV genotype 1 (n = 15)	Control genotype 1 (n = 5)	HCV genotype 3 (n = 22)	Control genotype 3 (n = 5)
Gender (male/female)	8/7	2/3	10/12	2/3

Age (years)	48.1 ± 12.6	49.3 ± 10.3	37.5 ± 9.5	49.3 ± 10.3

ALT U/l)	80.2 ± 69.3	61.4 ± 40.9	40.5 ± 34.2	61.4 ± 40.9

AST (U/l)	76.7 ± 67.6	37.4 ± 17.6	58.4 ± 32.1	37.4 ± 17.6

γ-GT (U/l)	133.7 ± 23.3	60 ± 29.8	97.8 ± 10.6	60 ± 29.8

Total bilirubin (mg/dl)	1.4 ± 0.2	0.7 ± 0.2	0.9 ± 0.5	0.7 ± 0.2

Prothrombin time (%)	103 ± 11.2	108.6 ± 12.1	114 ± 9	108.6 ± 12.1

Triglycerides (mg/dl)	153.2 ± 94.3	137.6 ± 62.9	194.5 ± 86.2	137.6 ± 62.9

Cholesterol (mg/dl)	201.8 ± 52.5	201 ± 43.6	208.6 ± 37.2	201 ± 43.6

Virological response	11/4 (73.3%)	/	19/3 (86.3%)	/

Ascites	None	none	none	none

FibroScan (kPa) Pre-therapy	8.8 ± 5.4	9.8 ± 3.9	7.5 ± 1.9	8.2 ± 2.4

FibroScan (kPa) Post-therapy	7.4 ± 1.8	9.5 ± 3.3	6.2 ± 1.2	8.7 ± 2.9

Values are presented as means ± SD. Genotype 1 was present in 15 patients with hepatitis C whereas 22 patients had genotype 3. Additionally, a group of 10 subjects with untreated HCV was used as a control. No relationship was found between BIA measurements and laboratory data.

### Body weight is significantly reduced in patients with genotype 1 receiving antiviral treatment for 48 weeks

**Figure 2:**
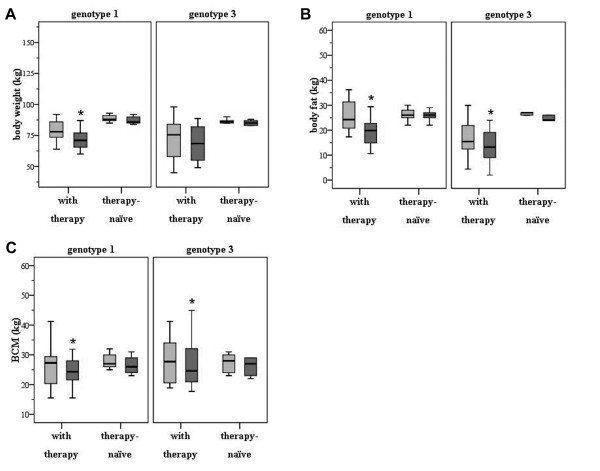
**(A) Body weight is significantly reduced in HCV patients with genotype 1 following 48 weeks of antiviral treatment**. No significant decline was present in the control group during the observation period. For all figures, the initial measurements are depicted as light grey and the follow-up measurements are depicted as dark grey blots. (B) Body fat is significantly decreased in HCV patients following antiviral regimens - irrespective of genotype or duration of therapy. No alterations were observed within the control group. (C) A significant reduction in body cell mass was also observed in both HCV groups post-therapy. Again, no significant alterations were present in the therapy-naïve group.

As demonstrated in Figure 2A, body weight significantly decreased in patients with genotype 1 following antiviral treatment for 48 weeks (78 ± 13.1 kg before therapy versus 71 ± 15.3 kg after therapy; p < 0.001). Body weight was also reduced in subjects with genotype 3 receiving antiviral medication for 24 weeks, though not statistically significant (75.5 ± 20.7 kg before therapy versus 68.5 ± 21 kg after therapy; n.s.). In contrast, almost no alterations in body weight were observed in the control group - irrespective of the genotype (genotype 1: 88.8 ± 3.1 kg at baseline, 87.4 ± 12.3 kg after 48 weeks; genotype 3: 86.6 ± 2.1 kg at baseline, 85.2 ± 2.2 kg after 24 weeks; n.s.).

### Body fat is significantly decreased in patients with hepatitis C following antiviral therapy

BF was decreased in patients with genotype 1 (24.2 ± 6.7 kg pre-therapy, 19.9 ± 6.6 kg post-therapy; p < 0.001; Figure 2B). Likewise, BF was decreased in patients with genotype 3 (15.4 ± 10.9 kg pre-therapy, 13.2 ± 12.1 kg post-therapy; p < 0.005). Interestingly, reduction in BF was more profound in genotype 1 following 48 weeks of therapy. However, no significant alterations in BF were observed within the therapy-naïve HCV groups - neither after 24 nor after 48 weeks (genotype 1: 26.2 ± 3.0 kg at baseline, 25.8 ± 2.5 kg after 48 weeks; genotype 3: 26.8 ± 2.8 kg at baseline, 25.6 ± 2.6 kg after 24 weeks; n.s.).

### Body cell mass is reduced in HCV patients after antiviral therapy

In HCV genotype 1 patients, BCM decreased from 27.3 ± 6.8 kg before antiviral treatment to 24.3 ± 7.2 kg (p = 0.02; Figure 2C). We also observed a significant reduction in BCM in patients with HCV genotype 3 (27.7 ± 8.8 kg before versus 24.6 ± 7.6 kg after treatment; p = 0.01). Again, no changes in BCM were observed in untreated HCV patients (for genotype 1: 28.0 ± 2.9 kg at baseline versus 26.6 ± 3.3 kg after 48 weeks and for genotype 3: 27.2 ± 3.5 kg at baseline versus 26.0 ± 3.3 kg after 24 weeks; p > 0.5).

### Determination of extracellular mass revealed no significant alterations in patients infected with hepatitis C following antiviral regimens

**Figure 3:**
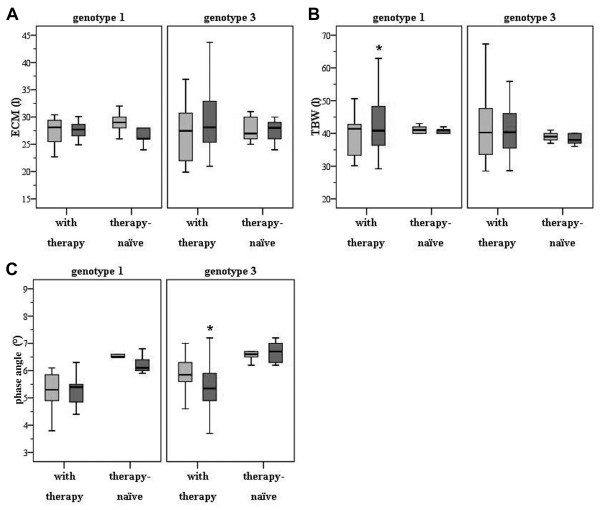
**(A) No significant changes in extracellular mass were detected in HCV patients related to genotype or duration of antiviral treatment**. (B) Total body water is significantly reduced in HCV-infected patients with genotype 1. As demonstrated, TBW decreased with the duration of antiviral therapy for 48 weeks. (C) Phase angle was significantly decreased in patients with genotype 3. Interestingly, no alterations in PA were present in patients with genotype 1 treated for 48 weeks.

As depicted in Figure 3A, ECM did not change in either HCV genotype 1 (28.1 ± 4.4 l before and 27.7 ± 5.2 l after therapy; p > 0.05) nor in HCV genotype 3 patients (27.4 ± 5.2 l before and 28.1 ± 6.0 l after therapy; p > 0.05). Similarly, no significant changes in ECM were detected within the untreated HCV cohort (for genotype 1: 29.0 ± 2.2 l at baseline versus 27.2 ± 3.0 l after 48 weeks and for genotype 3: 27.8 ± 2.5 l at baseline versus 27.4 ± 2.4 l after 24 weeks; p > 0.05).

### Total body water is significantly reduced in HCV patients with genotype 1 following antiviral treatment for 48 weeks

TBW was reduced in patients with genotype 1 following antiviral treatment for 48 weeks (41.4 ± 7.9 l pre-therapy vs. 40.8 ± 9.5 l post-therapy; p < 0.01; Figure 3B) whereas no significant alterations could be observed for HCV genotype 3 patients (40.3 ± 10 l pre-therapy vs. 40.4 ± 9.3 l post-therapy; n.s.). In addition, no significant changes for TBW were present in patients with untreated HCV infection (genotype 1: 41.2 ± 1.3 l at baseline, 40.8 ± 0.8 l after 48 weeks; genotype 3: 39.0 ± 1.5 l at baseline, 38.2 ± 1.7 l after 24 weeks; n.s.).

### BIA-derived phase angle is significantly decreased in HCV patients with genotype 3 following antiviral regimens

As shown in Figure 3C, PA did not differ before and after antiviral therapy in HCV patients with genotype 1 (5.3 ± 0.7° before therapy versus 5.4 ± 0.7° after therapy; p > 0.05) whereas in genotype 3 patients PA was significantly decreased (5.9 ± 0.7° before therapy versus 5.4 ± 0.8° after therapy; p < 0.001). Again, no changes were observed in patients with untreated hepatitis C (genotype 1: 6.5 ± 0.2° at baseline, 6.2 ± 0.3° after 48 weeks; genotype 3: 6.6 ± 0.3° at baseline, 6.6 ± 0.4° after 24 weeks; n.s.).

### Adverse effects of antiviral treatment are more prominent in HCV-infected patients with alterations in body composition

In a further sub-analysis we found a reduction in BF and BCM to a similar degree in both HCV genotypes following antiviral therapy - without any correlation to the recorded adverse effects of antiviral treatment (Table 2). Interestingly, a decrease in TBW was more often accompanied with episodes of fatigue and cephalgia in patients with genotype 1. Moreover, we observed that a decline in PA was more often associated with flu-like symptoms - as revealed for patients with genotype 3. We speculate that this may be related to a delayed dehydration in this cohort of patients.

**Table 2:** Percentage of adverse effects related to the genotypes and alterations in body composition following antiviral treatment.

Adverse effects	HCV genotype 1 (n = 15)	HCV genotype 3 (n = 22)
Cephalgia	8/15 (53.3%) *	8/22 (36.3%)

Fatigue	13/15 (86.6%) *	12/22 (54.5%)

Flu-like symptoms	10/15 (66.6%)	18/22 (81.8%) *

Symptoms of fatigue and cephalgia were more evident in patients with genotype 1 whereas flu-like symptoms were more present in patients with genotype 3 following antiviral treatment (* p < 0.05).

## Discussion

BIA has been used for the assessment of malnutrition in patients with liver cirrhosis. In this setting, use of BIA has been demonstrated to offer a considerable advantage over other widely available but less accurate methods like anthropometry or the creatinine approach [27]. Despite some limitations in patients with ascites, BIA is a reliable bedside tool for the determination of BCM in cirrhotic patients. Pirlich and colleagues, however, demonstrated that removal of ascites had only minor effects on BCM as assessed by BIA [28].

In a recently published study by Antaki et al., BIA was used for the evaluation of hepatic fibrosis in patients with chronic HCV infection [23]. The aim was to assess whether BIA can differentiate between minimal and advanced liver fibrosis in a cohort of 20 HCV-infected patients. The authors found no significant differences with respect to PA, R, or Xc for the whole body and the right upper quadrant measurements in any axes - irrespective if minimal or advanced fibrosis was present. Furthermore, Romero-Gomez and co-investigators found that in HCV patients infected by genotype 3a, hepatic steatosis correlated significantly with intrahepatic HCV-RNA load. However, in genotype 1, hepatic steatosis was associated with host factors such as leptin levels, BMI, percentage of BF, and visceral obesity [29]. Following antiviral treatment, we found a significant reduction in body fat in patients with genotype 3. Interestingly, major alterations in BMI were not present. We suggest a loss in fatty tissue, which might be compensated e.g. by increased water storage. Although we have no evidence for this mechanism, as we did not further investigate this issue. For clinical purpose, body fat comprises an intrinsic risk factor for diabetes, hyperlipidemia, NAFLD, and cardio-vascular diseases whereas a higher body cellular mass is not associated to known health risks. In addition, analyzing TBW by BMI method may further improve to predict a patient's hydration level while ECM contains the metabolically inactive parts of the body components including bone minerals and blood plasma. In a further cross-sectional analysis by Delgado-Borrego and colleagues comparing 39 HCV-positive with 60 HCV-negative orthotopic liver transplant (OLT) recipients, the authors found by BIA-derived measurements that HCV infection and BMI were independent predictors of insulin resistance (IR), respectively. HCV infection was associated with a 35% increase in IR [30].

The present study was conducted to investigate whether BIA can be used to monitor changes or alterations in body composition parameters in patients with chronic HCV infection following antiviral therapy for 24 or 48 weeks. Although compromised by the small sample size, our results suggest that bioelectrical impedance analysis does have the sensitivity required to distinguish significant differences in patients with chronic HCV infection with respect to body weight, BF, BCM, and TBW, in part related to the genotype. We also included a control group with untreated HCV infection whereas several studies of BIA in healthy subjects have shown mean PA values ranging from 6.3 to 8.2° [21,31]. Our findings for PA in untreated HCV patients did fall in that range. It should be noted that BIA can be affected by both BMI and age. A higher BMI is known to correlate with a higher PA, possibly secondary to the effect of adipose tissue on resistance [32]. Other studies have suggested a gradual decrease in PA with age [31,33]. Our results did not show a correlation between gender and age or biochemical and virologic response rates to PA (data not shown) in either group, probably due to the small sample size. However, to best of our knowledge this is the first study demonstrating alterations in body composition measured by BIA in patients with chronic HCV infection following antiviral treatment.

The identification of prognostic factors in patients infected with HCV is of considerable importance for the clinical management of this disease. The current study was performed to investigate whether BIA-derived phase angle or alterations in body composition can predict or monitor the outcome to antiviral therapy in HCV-infected patients. Our study demonstrates that a reduction in PA was clinically more often accompanied with episodes of flu-like syndromes in patients with genotype 3 whereas symptoms like fatigue and cephalgia were more evident after a decline in total body water in patients with genotype 1 (Table 2). This information would be helpful in patient management and may implicate that for example in patients with genotype 1 following antiviral treatment fluid support should be planned or modified whereas in genotype 3 flu-like symptoms should be treated earlier with e.g. acetaminophen. As a step to further understand the clinical applications of BIA-derived assessments, we propose that similar studies with larger sample sizes are needed to further validate the prognostic significance of PA and TBW determinations in patients infected with HCV. Investigations into other non-invasive modalities for the assessment of alterations in body composition in patients with hepatitis C infection should be pursued.

## Abbreviations

ALT: alanine aminotransferase; AST: aspartate aminotransferase; BCM: body cell mass; BF: body fat; BIA: bioelectrical impedance analysis; BMI: body mass index; ECM: extra cellular mass; HCV: hepatitis C virus; IFN-α: interferon-α; PA: phase angle; TBW: total body water

## Competing interests

The authors declare no conflict of interest.

## Authors' contributions

All authors read and approved the final manuscript.

AK designed the study, acquired clinical patient data, analyzed and interpreted the data, and drafted the manuscript. JH analyzed and interpreted the data, revised the manuscript for important intellectual content, and gave technical support on BIA measurements. MN performed BIA measurements. JE and AW acquired clinical data and assisted in statistical analysis. MP revised the manuscript for important intellectual content. GG obtained funding, gave administrative and material support, and supervised the study. AC designed the study, interpreted the data, revised the manuscript for important intellectual content, obtained funding, and supervised the study.
